# Overcoming Stigma in Women’s HIV and Syphilis Care: The Role of Faith in Healing

**DOI:** 10.5334/aogh.5042

**Published:** 2026-01-02

**Authors:** Irene A. Stafford, Phillip C. Johnson, Robin L. Beach

**Affiliations:** 1 Department of Obstetrics, Gynecology and Reproductive Sciences, McGovern Medical School, The University of Texas Health Science Center at Houston (UTHealth) Houston, Texas, USA; 2 Department of Internal Medicine, Division of Infectious Diseases, McGovern Medical School, UTHealth Houston, Houston, Texas, USA; 3 Department of Obstetrics, Gynecology and Reproductive Sciences, McGovern Medical School, UTHealth Houston, Houston, Texas, USA

**Keywords:** stigma, maternal health, HIV, syphilis, faith, global health, Guatemala

## Abstract

Point-of-care (POC) testing for syphilis and HIV is an effective way to provide same-day testing, results management, counseling, and treatment. Although commonly used in antenatal or sexual health clinics, our field study aimed to offer POC testing to women attending a standard care clinic in Guatemala. Nearly all women accepted testing, highlighting the patient-centered benefits and acceptability of screening in non-stigmatizing settings. Upon disclosure of results, especially positive diagnoses, patients drew comfort and resilience from their faith, an often-underprioritized resource not typically considered when addressing sexual health. These results underscore the value of integrating POC testing into routine care and reveal the important role of spirituality in how many patients and providers process diagnosis and illness.

Last September, in Puerto Barrios, Guatemala, I had one of those experiences that reshapes how you see the world. As a professor in Obstetrics and Gynecology at UTHealth, I led a small team including a resident, two medical students, and a college student, on a mission with Faith in Practice. Funded by a UT global health scholarship, we dove into research on curbing mother-to-child transmission of syphilis and HIV in resource-scarce settings. What started as a week of counseling hundreds of women on sexual health turned into a profound lesson in resilience, faith, and the quiet power of breaking silence.

We uncovered a heavy reality. Among the over 150 women we screened, syphilis and HIV rates hovered around 4%, a burden that echoes challenges in underserved US communities. But the numbers faded next to the stories. These infections carry a deep stigma including feelings of shame that keeps women from routine check-ups and open conversations about sexual health. Universally, this blocks the very basics of prevention, a simple test with a timely dose of penicillin for syphilis or antiretrovirals for HIV. We know these treatments are effective, reducing transmission risks by up to 90% when stigma does not stand in the way. Yet fear of judgment often does, turning primary care visits into missed opportunities for women, mothers, and babies.

When sharing the diagnosis of HIV or syphilis infection with these special women who broke through the fear and trusted us with their care, we encircled them with support. Through the tears, we counseled them that their bravery and trust in us was key to preventing the negative effects of undiagnosed disease.

But they taught us all way more.

They shared it was through their faith in God and belief that through prayer and the relationships built with us, that they would get through this new diagnosis. They were grateful to God for the path that led them to the Faith in Practice medical team that day.

Prayers before tests, grateful tears after plans for treatment. These were not just patients; they were pillars of strength; modeling how spiritual anchors can propel us toward care.

Religion does not have to be a barrier to sexual health; in many cases, including Puerto Barrios, it becomes a vital tool. Here, optimal care flowed through a faith-based program that might otherwise have been overlooked, weaving Christian principles of compassion and community into medical delivery.

We are most effective when we dismantle stigma through empathetic conversations and community trust. Better still when we embrace *all* sources of strength, including faith, as equally vital. This holistic, patient-centered approach is how we reduce maternal and congenital syphilis and HIV transmission, honoring both science and the whole person.

